# Regulation of skeletal muscle growth by the IGF1-Akt/PKB pathway: insights from genetic models

**DOI:** 10.1186/2044-5040-1-4

**Published:** 2011-01-24

**Authors:** Stefano Schiaffino, Cristina Mammucari

**Affiliations:** 1 Venetian Institute of Molecular Medicine (VIMM), Padova, Italy; 2 Department of Biomedical Sciences, University of Padova, Padova, Italy

## Abstract

A highly conserved signaling pathway involving insulin-like growth factor 1 (IGF1), and a cascade of intracellular components that mediate its effects, plays a major role in the regulation of skeletal muscle growth. A central component in this cascade is the kinase Akt, also called protein kinase B (PKB), which controls both protein synthesis, via the kinases mammalian target of rapamycin (mTOR) and glycogen synthase kinase 3β (GSK3β), and protein degradation, via the transcription factors of the FoxO family. In this paper, we review the composition and function of this pathway in skeletal muscle fibers, focusing on evidence obtained *in vivo *by transgenic and knockout models and by muscle transient transfection experiments. Although this pathway is essential for muscle growth during development and regeneration, its role in adult muscle response to mechanical load is less clear. A full understanding of the operation of this pathway could help to design molecularly targeted therapeutics aimed at preventing muscle wasting, which occurs in a variety of pathologic contexts and in the course of aging.

## Introduction

Muscle wasting occurs in a variety of conditions, such as cancer cachexia, diabetes, renal failure and heart failure, and aging itself. The survival and quality of life of these patients and of the older person can be improved by counteracting loss of muscle mass and strength, and different approaches to this have been explored, including nutritional supplementation, resistance training and anabolic drugs. Recent advances in understanding the mechanisms responsible for muscle atrophy may pave the way to new and perhaps more effective treatments.

During the past several years, experimental studies based on rigorous genetic approaches have started to dissect the signaling pathways involved in muscle-mass regulation. Although studies on cultured muscle cells have contributed to identify these pathways, definitive evidence of their physiological relevance can only be obtained using *in vivo *systems, when myofibers have a mature structure, and the integrity of the neuromuscular and musculoskeletal system is preserved.

Two *in vivo *genetic approaches have been used to understand how muscle mass is regulated. One is based on the generation of transgenic and knockout mice, in which expression of muscle regulatory genes is selectively modified. The potential of the traditional gene overexpression or deletion approaches has been fully exploited with the introduction of the Cre/loxP technique and the use of inducible transgenes, which allows for the modulation of gene expression specifically in muscle tissues and at different developmental stages. It is thus possible to distinguish between the effects on the regulation of muscle growth during development from the effects on the maintenance of muscle mass in adulthood. An alternative approach to address muscle-mass regulation in the adult is based on *in vivo *transfection of skeletal muscles by electroporation with plasmids coding for specific components of signaling pathways, or for mutants bearing constitutively active or dominant negative properties. Transfection with plasmids able to generate specific small interfering RNAs in muscle fibers is also increasingly being used as a loss-of-function model. The ability of various factors in preventing muscle atrophy can be explored by transfecting denervated muscles.

In this review, we discuss how *in vivo *studies based on these genetic models have contributed to define the role of a specific signaling pathway, the insulin-like growth factor 1-Akt/protein kinase B (IGF1-Akt/PKB) pathway, in muscle mass regulation. Various aspects of the role of this pathway in skeletal muscle have been previously discussed [1-3]. The IGF1-Akt1 pathway shares most of its components with the insulin-Akt2 pathway, and the two pathways intersect at various levels. For example, insulin can also bind the IGF1 receptor and IGF1 can bind to the insulin receptor; furthermore, hybrids between the IGF1 and insulin receptors are present in skeletal muscle. However, insulin is especially important in glucose homeostasis, whereas IGF1 is mostly active in muscle growth. In this review, we consider exclusively the role of this pathway on growth rather than on metabolism.

### Overview of the IGF1-Akt/PKB pathway

**Figure 1:**
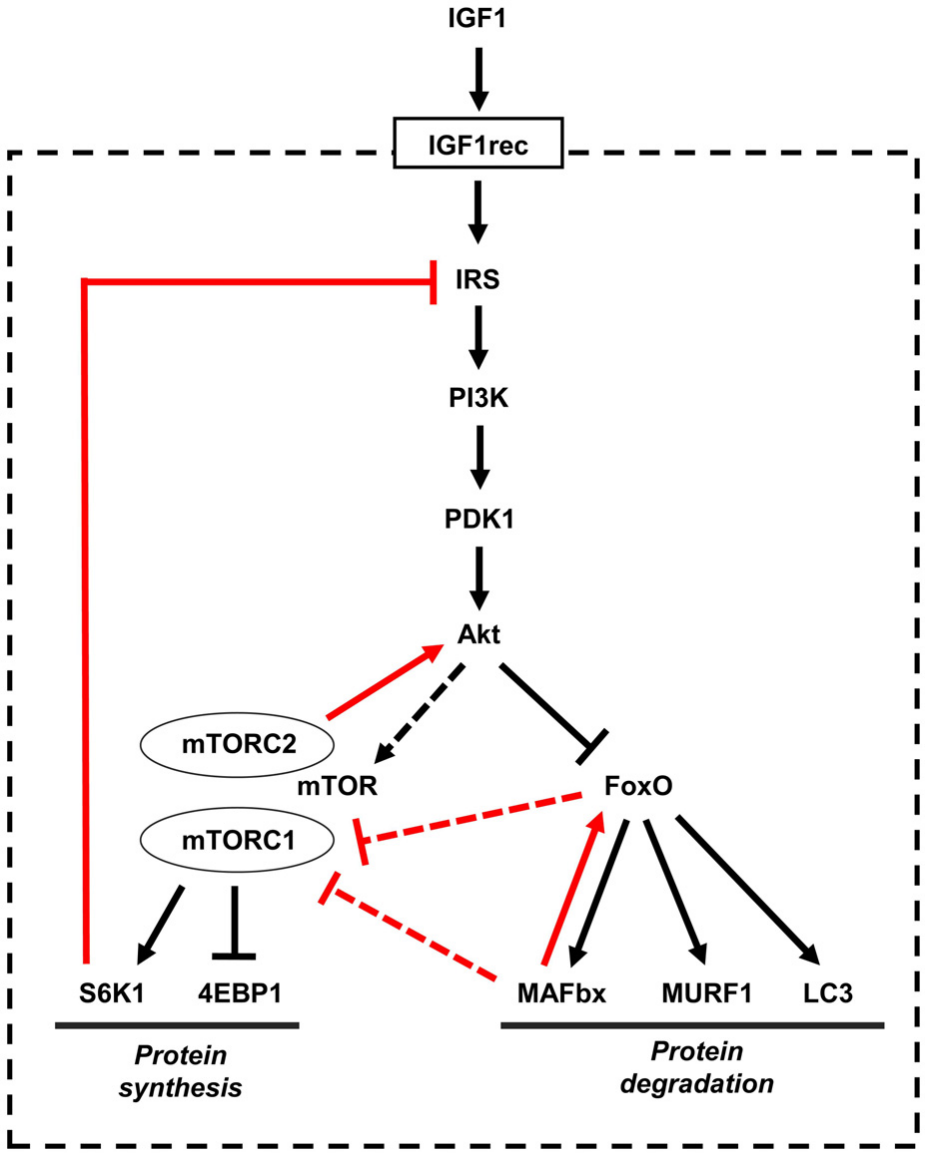
**The insulin-like growth factor 1 (IGF1)-Akt pathway controls muscle growth via mammalian target of rapamycin (mTOR) and FoxO**. The internal feedback loops that control the IGF1-Akt pathway are indicated in red. The dotted line indicates that the effect of Akt on mTOR is indirect, being mediated by the tuberous sclerosis complex (TSC) proteins 1 and 2 and by Rheb (Ras homolog enriched in brain). See text for details.

**Figure 2:**
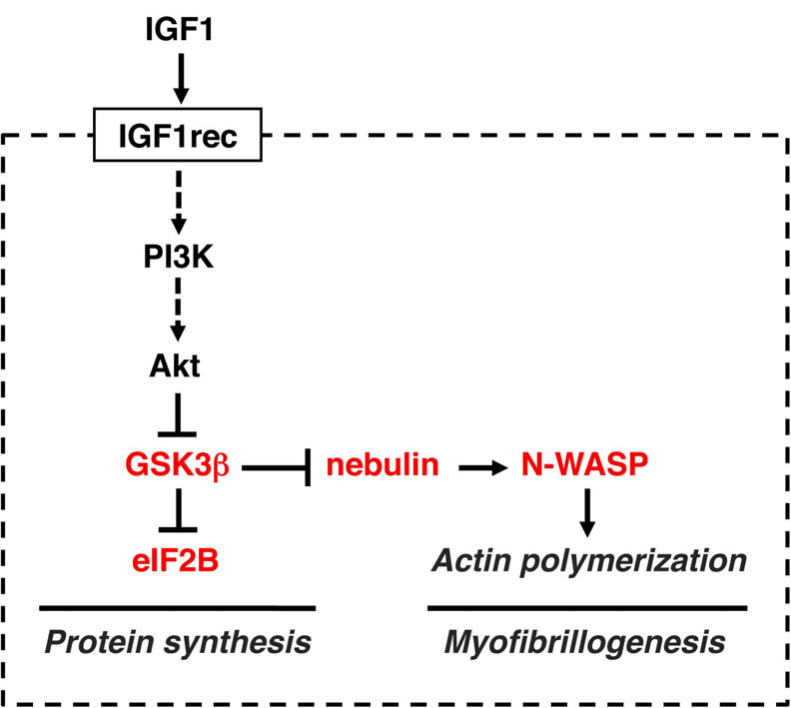
**The insulin-like growth factor 1 (IGF1)-Akt pathway controls muscle growth also via glycogen synthase kinase β (GSK3β)**. GSK3β inhibits protein synthesis via eukaryotic initiation factor 2B (eIF2B) and actin filament formation via nebulin and neuronal Wiscott-Aldrich syndrome protein (N-WASP). See text for details.

A simplified scheme of the IGF1-Akt pathway is shown in Figure 1. Binding of IGF1 to its receptor leads to activation of its intrinsic tyrosine kinase and autophosphorylation, thus generating docking sites for insulin receptor substrate (IRS), which is also phosphorylated by the IGF1 receptor. Phosphorylated IRS then acts as docking site to recruit and activate phosphatidylinositol-3-kinase (PI3K) which phosphorylates membrane phospholipids, generating phosphoinositide-3,4,5-triphosphate (PIP3) from phosphoinositide-4,5-biphosphate (PIP2). PIP3 acts in turn as a docking site for two kinases, phosphoinositide-dependent kinase 1 (PDK1) and Akt, and the subsequent phosphorylation of Akt at serine 308 by PDK1, leading to Akt activation. All these steps take place at the inner surface of the plasma membrane. Akt inhibits protein degradation by phosphorylating and thus repressing the transcription factors of the FoxO family, and stimulates protein synthesis via the mammalian target of rapamycin (mTOR) and glycogen synthase kinase 3β (GSK3β) [4]. FoxO factors are required for the transcriptional regulation of the ubiquitin ligases atrogin-1, also called muscle atrophy F-box (MAFbx) and muscle ring finger 1 (MuRF1), leading to the ubiquitylation of myosin and other muscle proteins (see below), and their degradation via the proteasome. FoxO factors are also required for the transcriptional regulation of the microtubule-associated protein 1 light chain 3 (LC3), which together with BCL2/adenovirus E1B interacting protein 3 (BNIP3) is essential for the activation of the autophagy-lysosome pathway. The effect of Akt on mTOR is indirect: Akt inhibits the tuberous sclerosis complex (TSC) proteins 1 and 2, which act as a GTPase activating protein (GAP) to inhibit the small G protein Ras homolog enriched in brain (Rheb) which activates mTOR signaling. mTOR forms two different protein complexes, the rapamycin-sensitive mTORC1, when bound to Raptor, and the rapamycin-insensitive mTORC2, when bound to Rictor [5]. TORC2 is required for Akt phosphorylation and activation [6]. mTORC1 phosphorylates S6 kinase (S6K), which in turn phosphorylates the ribosomal protein S6 and other factors involved in translation initiation and elongation, thus stimulating protein synthesis. TORC1 also activates eukaryotic translation initiation factor 4E (eIF4E) by phosphorylating the inhibitory eIF4E-binding proteins (4EBPs). Akt also promotes protein synthesis by phosphorylating and inactivating GSK3β, thus releasing the GSK3β-dependent inhibition of the eukariotic translation initiation factor 2B (eIF2B) (Figure 2).

The activity of the IGF1-Akt pathway is controlled by several feedback loops (Figure 1). Negative feedback involves S6K, which inhibits IRS by phosphorylation at multiple sites, thus inducing its degradation and altered cell localization [7]. Positive feedback involves mTORC2, which phosphorylates Akt at serine 473, a phosphorylation required for maximum activation of Akt in addition to phosphorylation at threonine 308 by PDK1 [8]. Rictor-dependent phosphorylation of Akt at serine 473 is required for Akt-mediated phosphorylation of FoxO but not of TSC2, thus it does not affect activation of S6K [5]. In another feed-forward mechanism, MAFbx, which is activated by FoxO, acts in turn as coactivator of FoxO [9]. MAFbx also appears to control protein synthesis by ubiquitylating and thus promoting the degradation of the eukariotic translation initiation factor 3 subunit F (eIF3F), thus suppressing S6K1 activation by mTOR [10,11]. FoxO factors have been shown to inhibit mTORC1 and activate Akt by inducing the expression of sestrin3 and Rictor in cultured mammalian cells [12], but this pathway has not been characterized in skeletal muscle *in vivo*.

**Figure 3:**
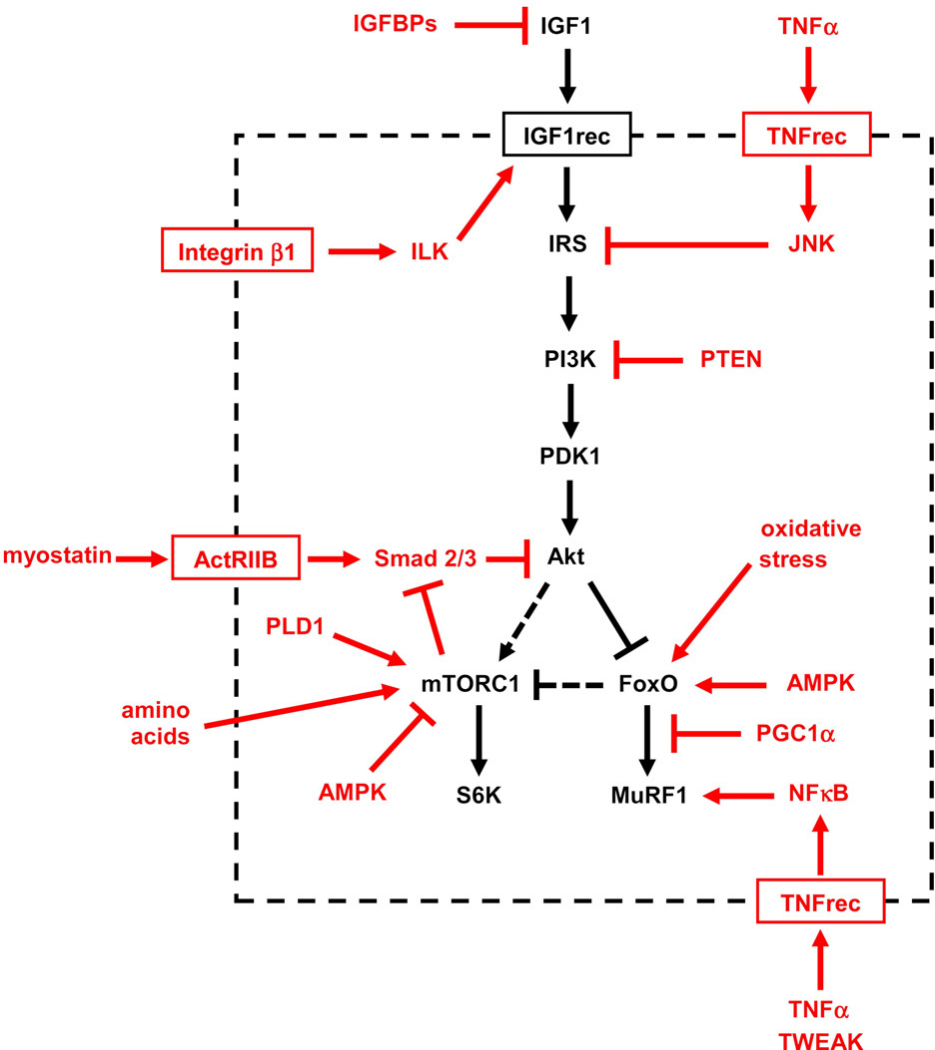
**Multiple factors and pathways affect insulin-like growth factor 1 (IGF1)-Akt signaling**. Various factors and pathways affecting the IGF1-Akt pathway are highlighted in red. See text for details.

The activity of the IGF1-Akt pathway can be modulated by a variety of other factors and pathways acting on different steps (Figure 3). IGF binding proteins (IGFBPs), the most important probably being IGFBP5, can block IGF1 action by inhibiting its binding to the IGF1 receptor. Mechanical signals via integrin β1 and integrin-linked kinase (ILK) lead to phosphorylation of the IGF1 receptor and activation of the PI3K-Akt pathway in muscle cells [13]. Whether another downstream integrin effector, the integrin-dependent focal adhesion kinase (FAK), affects the IGF1-Akt pathway in skeletal muscle remains to be established. FAK null mice are embryonic lethal, and skeletal muscle-specific conditional mutants have not been reported. Overexpression of FAK by electrotransfer was found to induce slight hypertrophy in adult rat muscles, but the effect on the IGF1-Akt pathway was not investigated [14]. Activation of IRS types is inhibited by phosphorylation of serine residues induced by inflammatory cytokines such as tumor necrosis factor α (TNFα) via Jun N-terminal kinase (JNK) [15]. PTEN (phosphatase and tensin homolog deleted from chromosome 10) is a lipid phosphatase that converts PIP3 to PIP2, thus opposing the action of PI3K, and interfering with Akt docking to the plasma membrane. Myostatin, also called growth and differentiation factor 8 (GDF8), acts as negative regulator of muscle growth, as shown by the hypertrophic phenotype induced by inactivation of the myostatin gene [16]. Myostatin, together with activin A, another member of the TGFβ family, acts via its receptor activin receptor IIB (ActRIIB) on Smad2 and Smad3, inhibitors of Akt; conversely, mTOR inhibits Smads [17,18]. In cultured muscle cells, addition of IGF1 dominantly blocks the effect of myostatin [17]. In adult skeletal muscle, muscle hypertrophy can be induced and muscle wasting prevented by blocking myostatin either via postdevelopmental myostatin gene knockout, or with follistatin (a myostatin antagonist) or anti-myostatin antibodies or a soluble ActRIIB decoy receptor (see [19,20] and references therein).

mTORC1 integrates growth factor signaling with a variety of signals from nutrients and cellular energy status. Direct activation of mTORC1 by amino acids is mediated by the Rag family of GTPases, which interact with Raptor [21,22]. By contrast, AMP-activated kinase (AMPK), an energy status sensor activated by ATP depletion, inhibits mTORC1 by phosphorylating and inactivating Raptor [23], and by phosphorylating and activating TSC2 [24]. The role of AMPK in the control of muscle fiber size through mTOR inhibition is demonstrated by the phenotype of AMPKα1 knockout and AMPKα1/α2 double knockout mice, which develop muscle hypertrophy [25,26]. mTOR can also be directly activated independently of PI3K/Akt signaling, by phosphatidic acid, generated by phospholipase D (PLD) in response to mechanical signals induced by eccentric muscle contractions [27-29].

AMPK can also impinge on the FoxO-dependent protein degradation pathways. AMPK can activate FoxO factors by phosphorylation at several regulatory sites distinct from Akt phosphorylation sites [30]. FoxO-dependent activation of atrogin-1 and muscle atrophy is inhibited by the peroxisome proliferator activated receptor γ coactivator 1α (PGC1α), a transcriptional coactivator. PGC1α transfection protects adult muscles from FoxO-dependent atrophy, and the higher levels of PGC1α in oxidative muscle fibers can explain their greater resistance to muscle atrophy [31]. Circulating cytokines, such as TNFα and TNF-related weak inducer of apoptosis (TWEAK), can activate MuRF1 via the transcription factor nuclear factor κB (NFκB) [32,33]. Finally, corticosteroids inhibit the IGF1-Akt pathway by acting at multiple levels, such as inducing decreased production of IGF1 and increased production of myostatin [34]. In addition, the glucocorticoid receptor and FoxO1 synergistically activate the MuRF1 gene [35].

Those listed above most probably represent only a minor proportion of the interactions of the IGF1-Akt pathway with other factors and pathways. Indeed, a recent interactome map based on a yeast two-hybrid screen for 33 components of the PI3K-mTOR pathway has identified 802 interactions, including 67 new validated interactions [36]. An additional complicating factor is the fact that various components of the IGF1-Akt pathway exist as multiple isoforms, many of which are coexpressed in skeletal muscle (Table 1). These include different splicing variants of IGF1, two types of IRS (IRS1 and IRS2), different isoforms of both the regulatory and catalytic subunits of PI3K, two types of Akt (Akt1 and Akt2) and S6K (S6K1 and S6K2), and several FoxO factors (FoxO1, FoxO3 and FoxO4). The different roles of some of these isoforms will be discussed below.

**Table 1:** Isoforms of major components of the IGF1-Akt pathway

Component	Isoforms	References
		

IGF1^1^	Two (mouse: 1A, 1B) or three (human: 1A, 1B & 1C) isoforms, differing in the C terminal peptide (E peptide); another two (mouse: 2A, 2B) or three (human: 2A, 2B & 2C) isoforms differing in signal peptide because of utilization of exon 2 instead of exon 1	[49]

IGF1 receptor	Heterotetramer made of two α-subunit (IGF-binding) and two β-subunit (tyrosine kinase). No isoforms, but hybrids with insulin receptor are present in skeletal muscle	[112]

IRS^2^	IRS1 and IRS2	[65,113]

PDK1^3^	No isoforms	[114]

PI3K^4 ^(class I)	Heterodimer of p85 regulatory and p110 catalytic subunits. p85: three isoforms (p85α, p55α, p50α) encoded by a single gene; two other isoforms (p85β, p55γ) coded by other genes. p55γ is not expressed in muscle. p110: three isoforms (p110α, p110β, p110δ). p110δ is not expressed in muscle	[115]

Akt/PKB^5^	Akt1/PKBα, Akt2/PKBβ & Akt3/PKBγ. Akt3 is not expressed in muscle	[4,116]

mTOR^6^	No isoforms, but mTOR can interact with different partners: Raptor in the rapamycin-sensitive complex mTORC1, or Rictor in the rapamycin-insensitive complex mTORC2	[5]

S6K^7^	S6K1 and S6K2	[117-119]

4EBP^8^	4EBP1, 4EBP2, 4EBP3. 4EBP3 is not expressed in muscle	[120]

FoxO	4 isoforms: FoxO1, FoxO3, FoxO4 and FoxO6. FoxO6 is not expressed in muscle	[121]

^1^Insulin-like growth factor.

^2^Insulin receptor substrate.

^3^3-Phosphoinositide-dependent kinase 1, coded by the *PDPK1 *gene (unfortunately the same abbreviation PDK1 is also used to indicate the pyruvate dehydrogenase kinase isoform 1, coded by the *PDK1 *gene).

^4^Phosphoinositide-3 kinase.

^5^Protein kinase B.

^6^Mammalian target of rapamycin.

^7^S6 kinase.

^8^Eukaryotic initiation factor 4E binding protein.

A large number of transgenic and knockout mouse models involving components of the IGF1-Akt pathway have been generated and are listed in Tables 2 and 3, respectively. *In vivo *transfection experiments leading to perturbation of IGF1-Akt pathway components in skeletal muscle are listed in Table 4.

**Table 2:** Transgenic models of the IGF1-Akt pathway: effect on growth

Genotype^1^	Viability	Phenotype	References
			

ASA-hIGF1	Viable	Muscle hypertrophy	[45,47]

MLC1-IGF1	Viable	Muscle hypertrophy	[51]

MCK-d.n. IGF1 receptor^2^	Viable	Transient delay of postnatal muscle growth; unaffected overload-induced muscle hypertrophy; impaired muscle regeneration	[59-62]

HSA-Akt1, inducible	Viable	Muscle hypertrophy	[79]

MCK-myrAkt1, inducible	Viable	Muscle hypertrophy	[80]

MLC1f-myrAkt1, inducible	Viable	Muscle hypertrophy	[82]

HSA-FoxO1	Viable	Muscle atrophy	[90]

^1^Promoters used to drive transgene expression: ASA = avian skeletal actin; HSA = human skeletal actin; MCK = muscle creatine kinase; MLC1 = myosin light chain 1 fast.

^2^This transgene acts as a dominant negative for both the insulin-like growth factor 1 receptor and the insulin receptor, thus causing diabetes.

**Table 3:** Knockout and knock-in models of IGF1-Akt pathway components: effect on growth

Genotype^1^	Viability	Growth phenotype	References
			

*Igf1 *null	Severe neonatal lethality	Severe growth retardation	[37-39]

*Igf1 *null in muscle (Mef2c-Cre)	Viable	Normal growth	[63]

*Igf1 *receptor null	Severe neonatal lethality	Severe growth retardation	[37,39]

*Igf1 *receptor null in muscle (Mef2c-Cre)	Viable	Reduced body weight, reduced muscle fiber number and size	[63]

*IRS1 *null	Viable	Reduced growth (weight 30-60% of control)	[64,122]

*IRS2 *null	Viable	Almost normal growth (birth weight 90% of control)	[65]

*PI3K p85α *+ *p55α *+ *p50α *null	Perinatal lethality		[66]

*PI3K p85α *+ *p55α *+ *p50α *null in heart & muscle (MCK-Cre)	Viable	Normal growth	[68]

*PI3K p85β *null	Viable	Normal growth	[67]

*PI3K p85α *+ *p55α *+ *p50α *null in heart & muscle (MCK-Cre) and *p85β *null	Viable	Reduced heart size but not muscle size	[68]

*PTEN *null in heart & muscle (MCK-Cre)	Viable	Cardiac hypertrophy but normal skeletal muscle growth; unaffected overload-induced muscle hypertrophy; improved muscle regeneration	[70,71,123]

*PDK1 *null	Embryonic lethality		[124]

*PDK1 *knock-in mutant unable to bind phosphoinositides	Viable	Reduced growth (weight 35% of control)	[125]

*PDK1 *null in heart & muscle (MCK-Cre)	Lethal at 5-11 weeks	Dilated cardiomyopathy but no change in muscle	[73]

*Akt1 *null	Viable but shorter life span	Mild growth retardation (weight 80% of control)	[74,75]

*Akt2 *null	Viable	Normal growth	[76]

*Akt1+Akt2 *null	Neonatal lethality	Severe growth retardation (birth weight 50% of control), marked muscle atrophy	[77]

*TSC1 *null	Embryonic lethality		[126]

*TSC2 *null	Embryonic lethality		[127]

*mTOR *null	Embryonic lethality		[128,129]

*mTOR *null in muscle (HSA-Cre)	Viable but premature death	Reduced postnatal growth due to reduced fast muscle growth, severe myopathy	[88]

*Raptor *null	Embryonic lethality		[6]

*Raptor *null in muscle (HSA-Cre)	Viable	Normal growth	[87]

*Rictor *null in muscle (HSA-Cre)	Viable	Reduced postnatal growth with severe myopathy and premature death	[87]

*S6K1 *null	Viable	Reduced growth (birth weight 80% of control), reduced muscle growth (fiber size 80% of control in adult mice)	[89,117]

*S6K2 *null	Viable	Normal growth	[130]

*S6K1+S6K2 *null	Perinatal lethality	Reduced growth	[130]

*4EBP1+4EBP2 *null	Viable	Normal growth	[131]

*FoxO1 *null	Embryonic lethality		[132,133]

*FoxO1 *null in muscle (HSA-Cre)	Viable	Normal growth, slow to fast switch in muscle	[134,135]

*FoxO3 *null	Viable but female sterility	Normal growth	[133,36]

*FoxO4 *null	Viable	Normal growth	[137]

*FoxO3+FoxO4 *null	Viable	Normal growth	[137]

*MAFbx *null	Viable	Reduced muscle atrophy after denervation	[92]

*Murf1 *null	Viable	Reduced muscle atrophy after denervation	[92]

^1 ^Promoters used to drive Cre recombinase expression: HSA = human skeletal actin; MCK = muscle creatine kinase; Mef2c = a promoter that lies 71 kb upstream of the first translated exon of the Mef2c gene and is sufficient to direct expression exclusively to skeletal muscle from embryonic day 8.5.

**Table 4:** In vivo transfection experiments leading to perturbation of the IGF1-Akt pathway in adult skeletal muscle^1^

Transgene	Perturbation	Effect	References
			

Igf1 (via virus)	Overexpression of Igf1	Muscle fiber hypertrophy (and muscle regeneration)	[50,106]

Igf1	Overexpression of Igf1	Prevention of glucocorticoid-induced muscle atrophy	[48,138]

RasV12C40 (Ras double mutant)	Activation of the PI3K-Akt pathway	Muscle fiber hypertrophy, which is blocked by rapamycin	[58,69]

c.a. PI3K^2^	Activation of PI3K	Muscle fiber hypertrophy	[69,78]

c.a. Akt	Activation of Akt	Muscle fiber hypertrophy, which is blocked by rapamycin	[69,78]

c.a. Akt (via virus)	Activation of Akt	Muscle fiber hypertrophy	[139]

c.a.FoxO3	Activation of FoxO3	Muscle fiber atrophy and activation of atrogin-1 reporter	[91]

Small interfering RNA to FoxO1 and FoxO3	Knockdown of FoxO1 and FoxO3 by RNA interference	Prevention of atrogin-1 reporter upregulation induced by starvation	[91]

Rheb^3^	Activation of Rheb and mTORC1	Muscle fiber hypertrophy	[140]

Small interfering RNA to N-WASP	Knockdown of N-WASP^4 ^by RNA interference	Muscle fiber atrophy	[86]

^1^Experiments based on intramuscular injection of plasmid DNA or viral vector or small interfering RNA expression vector. Injection of plasmid DNA or siRNA expression vector was followed by electroporation.

^2^Constitutively active PI3K.

^3^Ras homolog enriched in brain

^4^Neuronal Wiscott-Aldrich syndrome protein (N-WASP).

### IGF1 and IGF1 receptor

IGF1 was initially considered purely as a circulating growth factor produced by the liver and mediating the effect of growth hormone on body growth. However, subsequent studies showed that IGF1 is also expressed locally in many tissues, including skeletal muscle, suggesting that autocrine/paracrine effects of local IGF1 may be a major mechanism controlling tissue growth. *Igf1 *null mice exhibit severe growth retardation and most die soon after birth [37-39], whereas targeted ablation experiments showed that liver-derived IGF1, although it is the principal source of IGF1 in the serum, is not required for postnatal body growth [40,41]. However, this interpretation was challenged by the demonstration that conditional expression of IGF1 in the liver of an *Igf1 *null background contributes to about 30% of the adult body size [42]. This discrepancy was explained by the fact that a residual fraction of circulating IGF1 is detected in the liver-specific knockout [40,41] because of incomplete *Igf1 *gene excision [43]. In a similar study, elevated serum concentrations of IGF1, induced by overexpressing a rat *Igf1 *transgene specifically in the liver of *Igf1 *null mice, were able to rescue the severe growth-retarded phenotype observed in the*se *mice [44]. To define the effects of IGF1 produced by muscle cells, a transgenic construct was generated in which expression of a human IGF1 cDNA was driven by the avian skeletal α-actin gene [45]. IGF1 concentration in the serum was similar in wild-type and transgenic mice, but transgenic mice developed skeletal muscle hypertrophy [45-47]. In these mice, IGF1 overexpression was not sufficient to prevent the decrease in muscle mass induced by hind-limb unloading [46]. However, glucocorticoid-induced muscle atrophy was prevented by IGF1 overexpression via electroporation in adult rats [48].

The *Igf1 *gene can produce multiple transcripts by alternative RNA processing, thus generating divergent peptides at the carboxyl terminus, called the E peptides. Two isoforms, IGF1A and IGF1B, are found in most mammals, and an additional form, IGF1C, is present in primates and humans [49]. The E peptides appear to be essential for muscle growth regulation because viral delivery of IGF1A or IGF1B promoted functional hypertrophy in mouse muscles, whereas delivery of mature IGF1 devoid of E peptide failed to cause an increase in muscle mass [50]. The effect of an IGF1 isoform (IGF1A) was investigated in a transgenic mouse model, in which expression of this isoform was driven by the myosin light chain 1 fast promoter [51]. The transgenic mice showed postnatal increase in muscle mass and strength, and were protected from age-related muscle atrophy and weakness. Moreover, aging transgenic muscles retained a regenerative capacity comparable with that of young animals. Surprisingly, Akt activation was not detected in these mice [52], suggesting that IGF1 may signal via alternative pathways. A potential alternative pathway could be the serum- and glucocorticoid-responsive kinase 1 (SGK1), a PI3K-dependent kinase with structural homology to Akt, which is strongly expressed in many tissues, including skeletal muscle and heart [53]. SGK1 is activated by IGF1, PI3K and PDK1, and can induce phosphorylation of S6K and GSK3β in cardiomyocytes [54] and phosphorylation of FoxO3 in various cell types [55]. However, this alternative pathway has not been explored in skeletal muscle. IGF1-dependent signaling via the mitogen-activated protein kinase/extracellular signal-regulated receptor kinase (MAPK/ERK) pathway has also been implicated in IGF1-dependent muscle growth regulation [56,57], however a constitutively active Ras double mutant that selectively activates the ERK pathway did not induce hypertrophy of regenerating muscle fibers [58].

Until recently, the only available loss-of-function model for IGF1 and IGF1 receptor was a dominant-negative, kinase-inactive form of the β-subunit of IGF1 receptor driven by a muscle-specific promoter (MKR mice) [59]. Formation of a hybrid of the mutated IGF1 receptor with the endogenous IGF1 and insulin receptors caused impaired insulin and IGF1 receptor signaling pathways, specifically in skeletal muscle. The main phenotype of these mice was the development of peripheral insulin resistance and type 2 diabetes [59]. Muscle growth was transiently delayed from birth to 3 weeks of age, but muscle mass and force was normal in the adult [60]. Compensatory hypertrophy of the plantaris muscle after ablation of the synergistic gastrocnemius muscle, and associated Akt and S6K activation, were unaffected in these mice [61], but muscle regeneration was impaired [62]. More recently, specific loss-of-function mouse models have been generated. Muscle-specific knockout of the *Igf1 *receptor gene caused impaired skeletal muscle development with reduction in myofiber number and area and reduced numbers of type 1 fibers in gastrocnemius muscle [63]. By contrast, mice lacking *Igf1 *had no obvious phenotype, and their body weights were indistinguishable from control littermates at all postnatal times (reported as 'data not shown' in [63]).

### IRS, PI3K and PDK1

*IRS1 *null mice have much reduced growth (30-60% of control) [39,64], whereas *IRS2 *null mice have almost normal growth (about 90% of control), but develop type 2 diabetes [65], therefore IRS1 is thought to be downstream of the IGF1 receptor, and IRS2 downstream of the insulin receptor (Table 3). However, muscle-specific inactivation of the *IRS *genes has not yet been reported. *PI3K p85α *null mice have perinatal lethality, apparently due to liver necrosis [66], whereas *PI3K p85β *null mice are viable and have normal growth [67]. Conditional deletion of the *p85α *gene in skeletal muscle and heart showed normal growth [68], but when these mice were crossed with *p85β *null mice, the resulting double-mutant animals showed reduced heart but not reduced skeletal muscle size [68]. By contrast, constitutively active PI3K was found to induce muscle hypertrophy when transfected into regenerating skeletal muscle [69]. Conditional deletion of the *PTEN *gene in skeletal muscle and heart leads to hypertrophy of cardiac but not skeletal muscle. Overload-induced skeletal muscle hypertrophy is also unaffected by *PTEN *knockout [70], but maturation of regenerating muscle fibers is accelerated [71]. A skeletal muscle- and cardiac-enriched microRNA, miR-486, has been shown to target and inhibit the PI3K inhibitor PTEN, thus activating the Akt pathway [72]. Expression of miR-486 is regulated by myocardin-related transcription factor A (MRTF-A) and serum response factor (SRF). However, a role of miR-486 on muscle growth regulation has not yet been documented by *in vivo *genetic approaches. Conditional deletion of *PDK1 *in heart and muscle leads to dilated cardiomyopathy but no apparent change in muscle [73]. Thus, genetic evidence, based on loss-of-function approaches, supporting a role for PI3K and PDK1 in skeletal muscle growth is still missing.

### Akt/PKB

**Figure 4:**
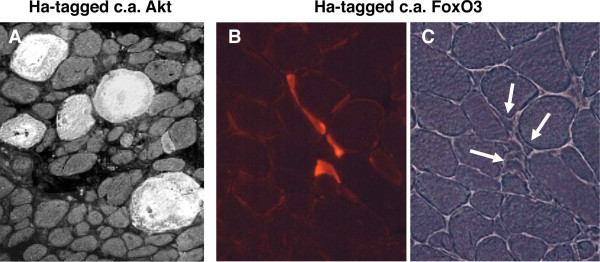
**Myofiber hypertrophy or atrophy induced by transfection of skeletal muscle with mutants of Akt or FoxO**. **(A) **Regenerating rat soleus muscle transfected with plasmid coding for constitutively active Akt1 linked to a hemagglutinin (HA) epitope. Muscle examined 7 days after transfection; section stained for the HA tag. Note the striking hypertrophy of labeled myofibers compared with untransfected neighbouring fibers. Modified from [69]. **(B,C) **Adult mouse tibialis anterior muscle transfected by electroporation with plasmid coding for constitutively active Ha-tagged FoxO3. Muscle examined 14 days after transfection; section stained for the HA tag. Note the striking atrophy of labeled myofibers compared with untransfected neighboring fibers. A phase-contrast image of the same field is shown in the right panel. Modified from [91].

Disruption of the *Akt1 *gene causes growth retardation and apoptosis [74,75], whereas deletion of *Akt2 *causes defects in glucose metabolism but not altered growth [76]. When both *Akt1 *and *Akt2 *genes were deleted, skeletal muscle atrophy at embryonic day 18.5 was observed, together with dwarfism, impaired skin and bone development, and reduced adipogenesis [77]. The striking effect of Akt1 on muscle size was demonstrated by *in vivo *transient transfection of a constitutively active Akt1 [69,78] (Figure 4A), and by transgenic mice overexpressing a constitutively active inducible Akt1 transgene in skeletal muscles [79-82]. Muscle hypertrophy was rapidly achieved in all cases when Akt1 expression was induced in the adult animal for a period ranging from 1 to 3 weeks. Downstream mediators of protein synthesis (S6K, S6) were activated, but no incorporation of satellite cells was observed [82]. Akt1 hypertrophic muscles showed increased strength, demonstrating that a functional hypertrophy was induced [80,82]. Moreover, muscle mass was completely preserved in denervated transgenic Akt mice [18]. Akt activation causes multiple changes in muscle gene expression, documented by microarray analyses [80,82,83]. The effects of Akt on muscle mass regulation can be mediated by several different downstream effectors, including GSK3β, mTOR and FoxO.

### GSK3β

A dominant-negative form of GSK3β has been shown to induce hypertrophy in skeletal myotubes [84], and overexpression of wild-type GSK3β in the heart induces a 30% decrease in heart size [85]. However, *in vivo *studies with GSK3β mutants in skeletal muscle have not been reported. A novel mechanism mediating the effect of GSK3β on muscle growth has been described recently [86]. GSK3β is able to phosphorylate nebulin at two Ser sites in the C-terminal region of nebulin localized to the Z-disk, thus preventing the interaction of nebulin with neuronal Wiscott-Aldrich syndrome protein (N-WASP), a ubiquitously expressed member of the WASP family, which is involved in actin assembly (Figure 2). IGF1-Akt signaling, by inhibiting GSK3β, allows the interaction of N-WASP with the unphosphorylated nebulin; the consequent recruitment of N-WASP to the Z-disk promotes actin nucleation and elongation of actin filaments. This process appears to be relevant to muscle growth *in vivo*, because long-term knockdown of N-WASP in adult mouse muscles causes atrophy of the transfected muscle fibers [86]. It was therefore suggested that IGF1-Akt signaling controls myofibril growth and maintenance via the GSK3β-nebulin-N-WASP pathway.

### mTOR

mTOR is part of two multiprotein complexes, TORC1, which contains contains Raptor and TORC2, which contains Rictor. To define the role of mTOR complexes in skeletal muscle, mice with muscle-specific ablation of *Raptor *and *Rictor *were generated [87]. Muscles from *Rictor *knockout mice were indistinguishable from wild-type controls. By contrast, Raptor-deficient muscles became progressively dystrophic, and were impaired in their oxidative capacity [87]. These changes were accompanied by decreased muscle force and downregulation of genes involved in mitochondrial biogenesis, including PGC-1α. A similar, although not identical, phenotype was observed in muscle-specific mTOR-deficient mice [88]. Like *Raptor *knockouts, mTOR-deficient muscles develop a myopathy reminiscent of muscular dystrophy, together with impaired oxidative metabolism. The myopathy was more severe in *mTOR *knockout than in *Raptor *knockout mice, possibly due to reduction in the content of dystrophin and other components of the dystrophin-glycoprotein complex. It was suggested that mTOR directly controls dystrophin transcription in a rapamycin- and kinase-independent manner [88].

Raptor and Rictor have different susceptibilities to rapamycin treatment, with Raptor activity being blocked by rapamycin, whereas Rictor activity is not. Nevertheless, Raptor inhibition and rapamycin treatment do not cause the same effect on muscles. Rapamycin blocks muscle growth in regenerating or Akt transfected muscles [69,78] but does not cause atrophy or dystrophy in adult mice treated for 15 days [18]. mTOR controls protein synthesis via different targets, including S6K, which exists in two isoforms, S6K1 and S6K2. *S6K1 *and *S6K2 *double-knockout mice have reduced muscle fiber size with unchanged number of myonuclei [89]. Deletion of *S6K1 *is sufficient to reproduce this atrophic phenotype.

### FoxO

The transcription factors of the FoxO family have emerged as major regulators of the muscle atrophy program. Mice overexpressing FoxO1 under a muscle-specific promoter have muscle atrophy and increased levels of the lysosomal protease cathepsin L [90]. FoxO3 is induced during muscle atrophy, and its overexpression is able to reduce muscle mass *in vivo*: the striking effect of a constitutively active mutant of FoxO3 when transfected into skeletal muscle is illustrated in Figure 4(B,C). By contrast, expression of dominant-negative FoxO3 inhibits dexametasone-induced muscle atrophy [91]. FoxO3 acts on the two major pathways of muscle protein degradation, the proteasomal and the autophagic-lysosomal pathways.

Direct evidence for FoxO-dependent activation of the ubiquitin-proteasome pathway was obtained with the demonstration that the muscle-specific ubiquitin ligases atrogin-1/MAFbx and MuRF1, which are induced in various models of muscle atrophy [92,93], are transcriptional targets of FoxO factors, and are upregulated by FoxO3 transfection in adult muscle [91,94]. Their role in muscle-mass regulation is supported by the finding that muscle atrophy induced by denervation is partially prevented in both *MuRF1 *null and *atrogin1/MAFbx *null mice [92]. MuRF1 is involved in the degradation of myosin heavy chains and other thick filament proteins, such as myosin light chains and myosin-binding protein C [95,96]. MuRF1 belongs to the muscle-specific RING finger protein family, which also includes MuRF2 and MuRF3. A redundant function of the different MuRFs is suggested by the finding that mice lacking a single MuRF gene do not have a striking skeletal muscle phenotype [92,97,98]. By contrast, *MuRF1 *and *3 *double-knockout mice develop skeletal and cardiac muscle myopathy with myosin accumulation [99], and *MuRF1 *and *2 *double-knockout mice have mild skeletal muscle hypertrophy and have reduced muscle loss during aging [100].

The role of FoxO3 as an inducer of autophagy is supported by the finding that transfection of adult muscle fibers with constitutively active FoxO3 causes accumulation of autophagic vacuoles, whereas fasting-induced autophagy is blocked by dominant-negative FoxO3 and by RNA interference-mediated FoxO3 knockdown [81,101]. FoxO3 is required for the upregulation of autophagy-related genes, such a *LC3 *and *Bnip3 *[81]. Inhibition of autophagy by muscle-specific knockout of the autophagy gene *Atg7 *was found to cause muscle atrophy, accompanied by a decrease in muscle force with accumulation of altered mitochondria and aberrant concentric membranous structures [102]. Moreover, inhibition of autophagy exacerbated muscle loss during denervation and fasting, suggesting that the persistence of dysfunctional organelles affects the progression of muscle atrophy [102]. The normal operation of the autophagic machinery is thus required for the disposal of altered cell organelles and the maintenance of muscle fiber integrity. An unexpected result was the finding that mTOR, which is considered as a major regulator of autophagy in different cell systems, does not appear to play a major role in muscle autophagy, as treatment with the mTOR inhibitor rapamycin has a limited effect on autophagy in skeletal muscle both *in vitro *and *in vivo *[81,101].

### Open questions

Several open questions on the role of the IGF1-Akt pathway in skeletal muscle remain to be answered, including the role of different isoforms, such as the IGF1 and the FoxO isoforms. Two general points that need to be addressed in future studies will be briefly considered here.

#### The role of the IGF1-Akt pathway in adult skeletal muscle

There is no doubt that IGF1 is a major regulator of muscle mass during development, thanks to its effect on myogenic cell proliferation and differentiation [103]. As to the role of IGF1 in adult skeletal muscle, several studies indicate that IGF1 can induce hypertrophy and block atrophy. In adult rats, local infusion of recombinant IGF1 results in muscle hypertrophy [104,105] and plasmid-mediated IGF1 gene transfer prevents corticosteroid-induced muscle atrophy [48]. In adult mice, virus-mediated IGF1 gene transfer results in muscle hypertrophy and prevents aging-dependent loss in muscle mass and force [50,106]. However, it is not clear whether IGF1 is involved in mediating the effect of load on adult muscle mass. Overexpression of IGF1 in the skeletal muscle of transgenic mice did not prevent unloading-induced muscle atrophy [46], although IGF1 transgene expression was decreased by unloading in these experiments. Overload hypertrophy was also unchanged in transgenic mice overexpressing a dominant-negative form of the IGF1 receptor [61], although interpretation of this model is complicated by the double effect of the transgene on both insulin and IGF1 signaling. The pathways mediating the effect of unloading and overloading on muscle size have not been identified, and as discussed above, it is possible that the Akt-mTOR pathway is involved either via integrin-ILK or via a direct effect on mTOR.

The role of IGF1 and its downstream effectors in adult skeletal muscle cannot be determined using traditional transgenic and knockout approaches, because developing muscles have greater plasticity than adult muscles, thus compensatory adaptations might occur in response to gene overexpression or ablation that are not seen when the genetic perturbation is induced in adult animals. This might account for the lack of a muscle phenotype in certain knockout models, for example, in the *PI3K *and *PDK1 *knockouts. Other knockout models cause maladaptative changes leading to muscle dystrophy rather than atrophy, such as that seen in the *mTOR *and *Raptor *knockout models. An understanding of the effect of genetic perturbation in adult rather than developing muscle is especially important for the identification of therapeutic targets and the design of countermeasures to prevent muscle wasting. To obtain useful information in this respect, it is essential to use inducible transgenic approaches, in which transgene overexpression or gene knockout is induced in adult animals. However, to our knowledge, the Akt inducible model is the only inducible model developed to date for the study of the IGF1-Akt pathway. Alternatively, *in vivo *transfection procedures can be used to explore the effect of transgene overexpression or gene knockdown by RNAi in adult muscles.

#### The effect of IGF1-Akt pathway activation on satellite cells

Muscle growth and muscle regeneration require the participation of satellite cells, and other cell types, including cells of blood vessels and, in the case of regeneration, inflammatory cells. This aspect must be considered in genetic models involving overproduction of IGF1, as this growth factor is known to act on different cell types, because of the presence of the IGF1 receptor in satellite cells and non-muscle cells. The effect on satellite cells is especially important for muscle hypertrophy. Satellite cells were reported to play a crucial role in the hypertrophic response induced by viral-mediated gene transfer of IGF1 in adult mouse muscles, as shown by the fact that gamma-irradiation, used to block satellite-cell proliferation, was found to reduce the hypertrophic effect of IGF1 overexpression [107]. However, interpretation of this experiment is complicated by the possibility that the reduced hypertrophic response may be due to effects of irradiation on myofiber protein synthesis [108], and perhaps on protein degradation as well. In another study on IGF1 transgenic mice, the finding that IGF1 overexpression causes first an increase in DNA, and only several weeks after birth an increase in protein mass, was interpreted as reflecting a primary effect on satellite-cell proliferation and fusion during the early postnatal stages, when satellite cells undergo active proliferation [47]. This interpretation would be consistent with the view that a growth stimulus is required for IGF1 to induce muscle hypertrophy *in vivo *[109]. However, satellite-cell proliferation was not directly examined in these studies. Satellite-cell proliferation and fusion leading to increase in myonuclei occurs during postnatal muscle growth [110] and during compensatory hypertrophy induced by ablation of synergistic muscles [111], as demonstrated by electron microscope autoradiographs after ^3^H-thymidine labeling. However, using an inducible transgenic model of muscle hypertrophy, no significant BrdU incorporation could be detected in satellite cells after short-term Akt activation in adult skeletal muscle, yet muscle hypertrophy was accompanied by increased force generation [82]. Thus, satellite-cell proliferation and fusion is not a prerequisite for a functional hypertrophy induced by Akt activation in adult skeletal muscle.

## Competing interests

The authors declare that they have no competing interests.

## Authors' contributions

SS conceived and designed the study. SS and CM drafted the manuscript.
